# Highly sensitive and specific detection of P-glycoprotein function for haematological and solid tumour cells using a novel nucleic acid stain.

**DOI:** 10.1038/bjc.1997.503

**Published:** 1997

**Authors:** H. J. Broxterman, G. J. Schuurhuis, J. Lankelma, J. W. Oberink, C. A. Eekman, A. M. Claessen, K. Hoekman, M. Poot, H. M. Pinedo

**Affiliations:** Department of Medical Oncology, Academisch Ziekenhuis Vrije Universiteit, Amsterdam, The Netherlands.

## Abstract

**Images:**


*British Journal of Cancer* (1997) **76**(8), 1029–1034
© 1997 Cancer Research Campaign

### Highly sensitive and specific detection of P-glycoprotein function for haematological and solid tumour cells using a novel nucleic acid stain

**HJ Broxterman[1], GJ Schuurhuis[2], J Lankelma[1], JW Oberink[2], CA Eekman[1], AME Claessen[3], K Hoekman[1], M Poot[4] and HM Pinedo[1]**

Departments of [1]Medical Oncology, [2]Hematology and [3]Pathology, Academisch Ziekenhuis Vrije Universiteit, Amsterdam, the Netherlands; [4]Molecular Probes, Eugene, OR, USA

**Summary** Progress in our understanding of the contribution of P-glycoprotein (P-gp)-mediated resistance to chemotherapy failure in haematological as well as solid tumours has been hampered by the lack of highly sensitive, reliable methods for the detection of P-gp function in fresh human tumour cells. The present study identifies the novel nucleic acid stain SYTO16 as a highly sensitive and specific dye to assess P-gp function. The effect of P-gp is expressed here as the ratio of dye fluorescence (RF) from cells incubated with dye with or without 2 μM of the P-gp inhibitor PSC 833. Using flow cytometric analysis, an RF of 0.9 was found for SYTO16 in the KB3-1 (P-gp$^-$) and 1.6 in KB8 (P-gp$^+$) cells. Three types of patients' cells were studied: (1) in haematopoietic CD34$^+$ cells, which are known to express P-gp, the RF was 6.0 for SYTO16 compared with 2.5 for rhodamine 123 and 1.3 for daunorubicin (mean of five individuals); (2) in acute myeloid leukaemia cells, the RF for SYTO16 was 1.0 in P-gp$^-$ and 4.5 in P-gp$^+$ samples; (3) for the first time, we have quantitated P-gp function in fresh human solid tumour (sarcoma) cells. We found, in a P-gp$^+$ leiomyosarcoma, an RF of 16 for SYTO16 and 2.7 for daunorubicin. This means that complete inhibition of P-gp function in these sarcoma cells would lead to an increase of daunorubicin accumulation with 170% compared with 30% in the CD34$^+$ cells. Next, we showed that SYTO16 could be fixed in nuclei by 3.6% formaldehyde treatment, allowing quantification of the nuclear fluorescence on cytospins by laser scanning microscopy. In conclusion, SYTO16 proved to have a combination of favourable properties: it can be excited at 488 nm and has large fluorescence enhancement upon binding to nucleic acids, allowing the use of low, non-toxic (< 10 nM) concentrations. Because the RF for SYTO16 is much higher than for daunorubicin, it can be applied for the determination of P-gp function in relatively small numbers of low-P-gp-expressing tumour cells by laser scanning microscopy. Individual sarcomas were found to have high P-gp function compared with CD34$^+$ cells. This assay may be used to select patients for P-gp modulation protocols.

**Keywords:** P-glycoprotein; fluorescence detection; DNA binding; CD34$^+$; sarcoma; PSC 833; SYTO16

The putative role of P-glycoprotein (P-gp) in chemotherapy resistance of human cancer has been studied extensively since P-gp was discovered (Juliano and Ling, 1976). In particular, the availability of monoclonal antibodies (Kartner et al, 1985; Scheper et al, 1988) and gene probes (Noonan et al, 1990) has greatly stimulated studies that have addressed the expression of P-gp in human tumour cells. Most of these studies correlate P-gp expression with clinical parameters, such as response to chemotherapy, duration of response or survival. Such studies are important because they may provide a rationale to adapt the treatment of patients predicted to have a poor response. In particular, the selection of patients for clinical trials with chemotherapy regimens aimed to circumvent or overcome the drug efflux by P-gp may be guided by measurements of P-gp levels in the patients' tumour cells. Progress in our understanding of the impact of P-gp on the response of cancer patients to chemotherapy is hampered by the inadequacy of current analytical methods to determine tumour cell P-gp in a quantitative, sensitive and reproducible way (Beck et al, 1996). A further improvement of immunocyto-/histochemical techniques to study low levels of P-gp expression is necessary (Beck et al, 1996; Broxterman et al, 1996*a*). An alternative approach to study the impact of P-gp expression is to measure the P-gp-mediated drug efflux in tumour cells. Such functional P-gp assays have been extensively used to assess drug transporter activity in cell lines and more recently in normal haematopoietic cells (Neyfakh et al, 1989; Chaudhary and Roninson, 1991) and haematological malignancies (Marie et al, 1993; Ross et al, 1993; Ino et al, 1994; Leith et al, 1995). These assays are based on the ability of P-gp to export fluorescent dyes, such as rhodamine 123, out of cells, allowing a convenient flow cytometric determination of P-gp activity. Whereas these fluorescent dyes are used to detect the P-gp activity in leukaemic cells (Broxterman et al, 1996 *a* and *b*), the specific problems with solid tumours, such as the difficulty to obtain single-cell suspensions leave this type of assay still largely unexplored (Broxterman et al, 1990; Kunikane et al, 1995). The lack of P-gp functional assays and of reliable immunohistochemical detection of P-gp has precluded definitive conclusions on the role of P-gp in solid tumours (Beck et al, 1996). Our purpose was to devise a sensitive functional P-gp assay, applicable to the study of different types of tumour cells. We describe the use of a novel nucleic acid stain that appears to be a highly sensitive and specific probe for P-gp function in haematological as well as solid tumour cells; as it can be excited at 488 nm and fixed in the cells, quantitative analysis of

*Received 16 January 1997*
*Revised 7 March 1997*
*Accepted 19 March 1997*

*Correspondence to:* HJ Broxterman, Vrije Universiteit, Department of Medical Oncology, BR 2.32, PO Box 7057, 1007 MB Amsterdam

**Table 1** Comparison of P-gp probes for human CD34+ and tumour cells

| | SYTO13 | SYTO16 | SYTO16 CLSM | Rho123 | Daunorubicin | $DiOC_2(3)$ |
|---|---|---|---|---|---|---|
| KB3-1 | 1.01 ± 0.19 | 0.90 ± 0.14 | 1.08 ± 0.12 | 0.98 ± 0.06 | 1.10 ± 0.06 | 1.05 ± 0.09 |
| KB8 | 1.79 ± 0.15 | 1.61 ± 0.26 | 1.98 ± 0.40 | 1.59 ± 0.20 | 1.17 ± 0.11 | 1.50 ± 0.16 |
| KB8-5 | 29.7 ± 16.6 | 19.6 ± 8.3 | – | 14.6-12.6 | – | – |
| $GLC_4$ | 1.1-1.0 | 1.1-0.9 | – | – | – | – |
| $GLC_4$/ADR | 0.9-0.9 | 0.8-0.9 | – | – | – | – |
| HL60 | 0.9 ± 0.2 | 1.0 ± 0.3 | – | – | – | – |
| HL60/ADR | 1.2 ± 0.6 | 1.1 ± 0.3 | – | – | – | – |
| CD34+ | – | 6.0 ± 2.2 | 4.0 ± 2.4 | 2.5 ± 0.5 | 1.3 ± 0.1 | – |
| AML1 (Pgp-) | – | 1.0 | 0.8 | 1.0 | 1.0 | – |
| AML2 (Pgp-) | 1.0 | 1.0 | 1.1 | 1.0 | 1.0 | – |
| AML3 (Pgp+) | – | 3.2 | 4.8 | 1.6 | 1.2 | – |
| AML4 (Pgp+) | 2.9 | 3.7 | 4.5 | 2.8 | 1.2 | – |
| Sarcoma 1 (Pgp+) | – | 16.0 | 10.7 | 4.9 | 2.7 | – |
| Sarcoma 1 (4°C, ON)[a] | – | 16.0 | – | 3.4 | 2.8 | – |
| Sarcoma 1 (thawed) | – | 21.1 | – | 5.6 | 2.5 | – |
| Sarcoma 2 (Pgp-) | – | 1.0 | 1.1 | 0.7 | 0.9 | – |
| Sarcoma 3 (Pgp+) | – | 6.0[c] | 3.9 | 2.5 | 1.5 | – |
| Sarcoma 3 (RPMI)[b] | – | 15.6 | – | 3.0 | 1.9 | – |

Cells were loaded with dyes with or without 2 μM PSC 833 for 75 min (daunorubicin, Rho123 or $DioC_2$ (3)) or for 45 min with SYTO probes. For $GLC_4$ and $GLC_4$/ADR, 1 mM probenecid was used as modulator (two experiments, separated by a hyphen). For HL60 and HL60/ADR, three different modulators were used (1 mM probenecid, 200 μM genistein and 2 μM PSC 833; the results were averaged for this cell line). Fluorescence was measured on a FACS Calibur, excitation at 488 nm, emission in FL1 or FL2 (daunorubicin) or by confocal laser scanning microscopy (CLSM). Data are ratios of mean fluorescence (RF) with or without modulator. For KB3-1, KB8 and KB8-5, the data represent two experiments (separated by a hyphen) or means ± s.d. from three to five independent experiments. For CD34+ cells, data are means ± s.d. from leucapheresis isolates of five different patients. For fresh tumour cells, data from individual samples are shown. P-gp expression was measured in fresh tumour cells with MRK-16 as described (Broxterman et al, 1996*b*). The ratio of the MRK-16/isotype control was 1.7 for both AMLs designated P-gp-, 7.9 and 9.0 for AMLs designated P-gp+ and 54, 1.6 and 12.7 (20.7 for cells in medium for 1 week) for the sarcoma cells labelled 1, 2 and 3 respectively. [a]ON, overnight. [b]RPMI, 1 week in medium. [c]For 80% of the cells.

P-gp function at the single-cell level is possible using a confocal laser scanning microscope (CLSM) equipped with an argon/krypton (Ar/Kr) laser.

### MATERIALS AND METHODS

### Cell lines

The human epidermoid carcinoma cell line KB3-1 and its P-gp-expressing sublines KB8 and KB8-5, which are about two and five times, respectively, more resistant to daunorubicin (DNR) than KB3-1, were cultured in Dulbecco's minimal essential medium (DMEM; Flow Labs, Irvine, UK) with 7.5% fetal calf serum (FCS; Gibco Europe, Paisley, UK). The $GLC_4$ and HL60 and their multidrug resistance protein (MRP)-overexpressing sublines $GLC_4$/ADR and HL60/ADR cells were cultured in RPMI-1640 + 10% FCS.

### Patient material

Three types of cells were studied, namely human peripheral CD34+ cells, acute myeloid leukaemia (AML) and freshly dissociated sarcoma cells. Leucapheresis samples were obtained from patients with haematological and oncological malignancies. Patients were treated with granulocyte colony-stimulating factor (G-CSF) and/or chemotherapy, resulting in increased numbers of CD34+ cells in peripheral blood. These CD34+ cells were isolated from leucapheresis samples after overnight storage at 4°C, using the MiniMacs System (Miltenyi Biotec, Bergisch Gladbach, Germany) according to the manufacturer's instructions. The purity was always > 90% CD34+, as determined by flow cytometric analysis. Blood samples obtained from patients with AML were subjected to Ficoll separation, and the nucleated cells were used immediately for the P-gp assays or frozen in liquid nitrogen and thawed as described (Broxterman et al, 1996*b*). Tumour tissue (10–15 g) from two leiomyosarcomas (sarcoma 1 and 3, Table 1) and one liposarcoma (sarcoma 2, Table 1) was dissociated immediately after resection by collagenase–DNAase I treatment, according to described methods (Broxterman et al, 1995). The resulting cell suspensions were used for P-gp analysis immediately after dissociation (3–4 h after tumour resection) or for comparison after overnight storage at 4°C (sarcoma 1), after freezing and thawing (sarcoma 1) or after keeping cells for 1 week in RPMI plus 20% FCS and glutamine at 37°C (sarcoma 3).

### Clonogenic assay

The clonogenic capacity of the CD34+ cells was assayed in semi-solid medium in the presence of 5% placenta-conditioned medium (3000–9000 cells per ml). Colonies (> 40 cells) and clusters (8–40 cells) were scored after 12 days. To study the toxicity of SYTO16, CD34+ cells were incubated for 45 min (the time chosen by us for dye loading in P-gp activity assays) with increasing concentrations of the dye and were washed and plated for 12 days in the clonogenic assay.

### Dyes

Daunorubicin was from Specia (Paris) and rhodamine 123 (Rho123) was from Sigma (St Louis, MO, USA). 3,3′-Diethyloxacarbocyanine iodide ($DiOC_2(3)$) and the thiazole orange derivatives SYTO13 [5 mM in dimethyl sulphoxide

*© Cancer Research Campaign 1997*

**Figure 1** Time course of SYTO16 accumulation in KB8 cells. Cells were incubated with 5 nM SYTO16 with or without 2 μM PSC 833 and, after indicated time points, the cells were washed and fluorescence was measured by flow cytometry. Data points are the mean of two determinations. A similar time curve was obtained for the KB3-1 and KB8-5 cells

(DMSO)] and SYTO16 (1 mM in DMSO) were from Molecular Probes (Eugene, OR, USA). Stock solutions of DNR (4 mM in 0.9% sodium chloride), Rho123 (1 mg $ml^{-1}$ in DMSO) and $DiOC_2(3)$ (0.1 mM in DMSO) were stored at – 20°C. PSC 833 was a gift from Sandoz (Basle, Switzerland) and was stored as 5 mM stock solution in ethanol.

### Dye accumulation

Cells were harvested and washed in accumulation medium (DMEM without bicarbonate and phenol red but with 20 mM Hepes and 10% FCS). About 0.5 × $10^6$ cells were incubated in eppendorf vials in 1 ml of accumulation medium with the dyes in the indicated concentrations with or without the P-gp inhibitor PSC 833 (2 μM). The vials were put in a 37°C water bath and carefully shaken. After the indicated time, the cells were immediately centrifuged and washed with 1.5 ml of cold accumulation medium, resuspended in 1 ml of medium and kept on ice until analysis. The cells were analysed by flow cytometry or by CLSM with image analysis. In the latter case, the cells were centrifuged for 5 min at 350 r.p.m. on slides precoated with 0.1% bovine serum albumin (about 35 000 KB cells per spin). Then the cells were fixed for 5 min with 3.6% formaldehyde solution, according to Willingham et al (1986). After washing with tap water, the spins were air dried horizontally in the dark and analysed immediately or stored in the dark overnight before analysis. In preliminary experiments, the results proved to be reproducible after storage for at least a week. On one occasion, KB8 cells were grown on a round coverslip (dimension Ø 24 mm) for one night and were then layered into accumulation medium in a temperature-controlled chamber (37°C), allowing the recording of real-time fluorescence in the cells during dye accumulation. For this experiment, cells were exposed to 25 nM SYTO16 for 1 h, followed by 2 μM PSC 833. After another hour, 3.6% formaldehyde solution was injected into the chamber for fixation of the cells.

### Flow cytometry

Fluorescence was analysed on a FACS Calibur (Becton Dickinson Medical Systems, Sharon, MA, USA). The fluorescence of 10 000 events was logarithmically measured at a laser excitation of 488 nm. The fluorescence of Rho123, $DiOC_2(3)$, SYTO13 and SYTO16 was collected at 530 nm (band width 30 nm) and of DNR at 585 nm (band width 42 nm). P-gp status was assessed with the monoclonal antibody MRK-16 (Dr T Tsuruo, Tokyo) and the FITC-labelled second antibody exactly as described (Broxterman et al, 1996*b*). The MRK-16 index is the ratio of the mean fluorescence of MRK-16-labelled cells divided by that of the isotype control antibody-labelled cells.

### Fluorescence microscopy

An inverted confocal laser scanning microscope (CLSM) TCS 4D (Leica, Heidelberg, Germany) equipped with an Ar/Kr laser and a 40 × /1.00–0.50 or 63 × /1.40 oil lens was used. Scanning, image processing and microscope control were performed using an OS9

© Cancer Research Campaign 1997

**Figure 2** Fluorescence in KB8 cells after incubation with 25 nM SYTO16. The cells were grown overnight on a coverslip and then put in a temperature-controlled chamber for real-time fluorescence measurements. (**A**) Background. (**B**) Steady-state accumulation (45 min). (**C**) After addition of 2 μM PSC 833. (**D**) 5 min after fixation with 3.6% formaldehyde

**Figure 3** Nuclear fluorescence per area of KB8 cells loaded for 45 min with different concentrations of SYTO16 with or without 2 μM PSC 833. After loading, the cells were centrifuged and cytospins fixed with 3.6% formaldehyde. The fluorescence values of about 200 nuclei per data point were measured minicomputer. Quantification of the images was performed with Leica Q500MC QWin software. The fluorescence of about 200 nuclei on 3 or 4 images from duplicate cytospins was recorded and quantitated. After subtraction of the background (from the area between the cells), the fluorescence of each nucleus was divided by the nuclear area and reported as mean fluorescence per area of about 200 cells.

### RESULTS AND DISCUSSION

The novel nucleic acid (DNA and RNA)-binding dyes SYTO13 and SYTO16 were tested in an attempt to identify sensitive and specific P-gp-activity probes applicable to flow cytometric as well as CLSM analysis of P-gp function. The SYTO dyes were chosen

**Figure 4** Nuclear SYTO16 fluorescence in the absence (**A**, **C** and **E**) or presence of PSC 833 (**B**, **D** and **F**) during dye loading in CD34+ haematopoietic cells (**E** and **F**) compared with KB3-1 (**A** and **B**) and KB8 (**C** and **D**) cells. Cells were cytocentrifuged after the appropriate labelling and fixed with 3.6% formaldehyde

 © Cancer Research Campaign 1997

because they exhibit a large increase in fluorescence upon nucleic acid binding and because they can be excited at the convenient wavelength of 488 nm. In addition, SYTO13 and SYTO16 have one positive charge at physiological pH.

### Flow cytometry

### Cell lines

Inhibition of P-gp with 2 μM PSC 833 induced a large increase in cellular SYTO13 and SYTO16 fluorescence in the P-gp-overexpressing KB8-5 cells. No increase of fluorescence was seen with the MRP inhibitor probenecid (1 mM; Feller et al, 1995*a*) in the $GLC_4$ and $GLC_4$/ADR cells. In the MRP-overexpressing HL60/ADR cells, we tested the two inhibitors probenecid and genistein (200 μM) and, in addition, 2 μM PSC 833, as this P-gp inhibitor also inhibits MRP-mediated drug transport in HL60/ADR but not in $GLC_4$/ADR (Feller et al, 1995*b*). No effect of these agents was seen in the HL60/ADR or the parental cell line (Table 1). These data show the specificity of SYTO13 and SYTO16 as substrates for P-gp.

More detailed investigations of the dyes were carried out using the KB8 cells, which is the cell line with the lowest P-gp overexpression available to us (Noonan et al, 1990). SYTO13 and SYTO16 behaved in a very similar way. The time course of SYTO16 accumulation in KB8 cells is shown in Figure 1. Steady-state accumulation of both dyes was reached within 30 min, with or without 2 μM PSC 833. The 45-min accumulation of both dyes was linear with the loading concentration being at least 1–25 nM (data not shown).

Next, we compared the sensitivity of SYTO13 and SYTO16 with fluorescent dyes that are commonly used for flow cytometric measurement of P-gp activity in leukaemias. Table 1 shows that the increase of fluorescence of the SYTO dyes in KB8 cells after P-gp inhibition with PSC 833 was of similar magnitude as that of Rho123 and $DiOC_2(3)$.

### Cells from patients

As the ratio of active to passive drug transport, which largely determines the net modulator effect, may be very different for different cell types (and is actually low for daunorubicin in the KB cells; Spoelstra et al, 1992), we applied the present test to some categories of cells for which it may be of value: normal human (CD34+) cells, AML blasts and freshly dissociated solid tumour (sarcoma) cells. Four AMLs were selected for P-gp expression based on the MRK-16 labelling index – two with MRK-16 index 1.7, which is a value associated with low or absent functional P-gp activity in AMLs as measured in a Rho123 test (Broxterman et al, 1996*b*), and two AMLs with a high MRK-16 index (7.9 and 9.0). It appeared that the modulation factor by PSC 833 in the P-gp+ human tumour samples and CD34+ cells was higher for SYTO16 than for Rho123 (Table 1). One of the leiomyosarcomas (sarcoma 1), which had a very high P-gp expression as measured with MRK-16 (see legend Table 1), also showed a large response to PSC 833, in particular when SYTO16 was used as P-gp substrate. The daunorubicin modulation of these cells was 2.7, which means that after P-gp inhibition these cells take up almost three times more daunorubicin. Such a large modulation has not been seen before, by us, in AML cells (Broxterman et al, 1996*b*), and the modulation was also much lower in the CD34+ cells. In addition, for this sarcoma, the P-gp function was compared in fresh cells, cells that had been stored overnight at 4°C and cells that were thawed after freezing in liquid nitrogen. As shown in Table 1, there was excellent agreement between the samples. A second leiomyosarcoma (sarcoma 3) had a P-gp activity similar to that of CD34+ cells, and a liposarcoma (sarcoma 2) was P-gp negative. In conclusion, these results show for the first time quantitative data on P-gp activity in fresh human solid tumour cells and allow an estimation of the impact of P-gp on drug accumulation in these tumours.

### Confocal laser scanning microscopy

### Cell lines

The flow cytometric method used to detect P-gp function established SYTO16 as a sensitive P-gp activity probe for solid tumours when a high yield of viable single cells is available. CLSM may be an alternative when only a small number of cells is available, provided that the method is sensitive and reliable. Therefore, we compared a CLSM method for the quantification of nuclear-fixed SYTO16 with flow cytometric data. We have previously shown that the low sensitivity of daunorubicin as a P-gp probe precluded the accurate measurement of the PSC 833 effect in KB8 cells, as well as in most AML samples, by CLSM analysis of about 200 cells (Broxterman et al, 1997). Using SYTO16 in KB8 cells, the development of fluorescence in individual cells was first followed qualitatively in real time before and after the addition of PSC 833 and after fixation. The increase in cellular SYTO16 fluorescence upon addition of PSC 833 was clearly visible (Figure 2). Moreover, after fixation, a large increase in nuclear fluorescence occurred, while the cytoplasmic fluorescence largely disappeared. To fully understand the biophysical nature of these changes, it would be necessary to study further the chemical interaction of SYTO16 with nucleic acids (DNA and RNA) under different conditions. Here, we take advantage of the fact that fixation of the dye allows a more practical assay. We found that the fluorescence was linear, at least within the range of concentrations of SYTO16 that are convenient to use (5–25 nM) (see Figure 3). In practice, we used a slightly higher concentration than for flow cytometry for which 1–5 nM was satisfactory).

### Cells from patients

To demonstrate some potential applications, we analysed P-gp function in CD34+ cells (an example is shown in Figure 4) and in AML samples (see Table 1). The ratios of the nuclear SYTO16 fluorescence with or without PSC 833 were 4.0 for CD34+ samples, 0.8 and 1.1 for the Pgp- and 4.8 and 4.5 for the Pgp+ AMLs. This method was also applied to the sarcoma cells. For example, in leiomyosarcoma 1, a PSC 833 effect on SYTO16 nuclear fluorescence of 10.7 was found by analysis of 204 cells without (mean nuclear fluorescence 5.2 ± 3.3) and 218 cells with PSC 833 (mean nuclear fluorescence 55.9 ± 16.3). The second leiomyosarcoma had a PSC 833 effect of 3.9 and the liposarcoma of 1.1 (Table 1). The results measured with the flow cytometer correlated well with the CLSM method. The CLSM method can be used for combined staining of a plasma membrane antigen with nuclear staining of the P-gp probe.

### Cytotoxicity assay

We determined the cytotoxicity of SYTO16 in a clonogenic assay for CD34+ cells from three individuals. No inhibition of clonogenicity was found at concentrations of SYTO16 up to 10 nM. Concentrations of 25 and 50 nM caused an inhibition of 20% and

© Cancer Research Campaign 1997

50% respectively. Thus, SYTO16 can be used at non-toxic concentrations for flow cytometric sorting of viable P-gp-expressing CD34+ cells, similar to Rho123 (Chaudhary and Roninson, 1991).

In summary, we have shown that SYTO16 is a highly sensitive probe for P-gp activity in a number of different experimental set-ups. In our present experiments, the sensitivity of the detection of P-gp function was higher than for Rho123 in the tumour samples. It is not clear why the difference in sensitivity between both probes for different types of cells (e.g. compare AML4 and sarcoma 3) is not the same, but factors related to passive membrane transport of the probes might play a role. Another P-gp probe used previously by us is calcein-AM (Feller et al, 1995*b*). As we found that the sensitivity was no higher for calcein-AM than for Rho123, we have not used it in the present comparison. Probes other than Rho123 may have advantages in certain experimental situations (e.g. $DiOC_2(3)$; Leith et al, 1995). It is important to realize that, for any new combination of probe and modulator, one should be aware of putative interactions with the fluorescence of the probe not related to pump activity and appropriate controls have to be performed. We have discussed the theoretical and practical considerations of the different probes in a more comprehensive way in Broxterman et al (1997).

Of importance, the present data show that the membrane integrity and the metabolic state of rapidly dissociated solid tumour cells is apparently sufficient to study P-gp activity. From previous experiments with human xenografts as a model (Broxterman et al, 1995), these results were expected, but they have now been shown for the first time in primary human tumour cells. In particular, the large modulation of SYTO16 fluorescence after P-gp inhibition, combined with the possibility to measure nuclear-fixed dye at an excitation wavelength of 488 nm, allows the reliable analysis of a relatively small number of tumour cells by CLSM. The concomitant analysis of P-gp activity and protein expression with monoclonal antibodies in the same single cells is possible. Clearly, the presupposition is that viable tumour cell suspensions are available or can be prepared; in certain carcinomas (breast), this may not be the case or more elaborate techniques may have to be used (Ljung et al, 1989; Dairkee et al, 1995).

In conclusion, we propose that the methodology described here to quantitate P-gp activity in normal as well as malignant blood cell populations and in solid tumour cells is superior because of its combination of simplicity and sensitivity. It may be used in clinical situations, such as the selection of patients for P-gp modulation protocols.

### ACKNOWLEDGEMENTS

This study is supported by the Dutch Cancer Society (KWF VU 93-626), the VSB fund and Bristol-Myers Squibb. Doke Wåhrer and Anita Stam provided excellent technical support.

### REFERENCES

Beck WT, Grogan TM, Willman CL, Cordon-Cardo C, Parham DM, Kuttesch JF, Andreeff M, Bates S, Boyett JM, Brophy N, Broxterman HJ, Chan HSL, Dalton WS, Dietel M, Fojo AT, Gascoyne R, Head D, Houghton PJ, Srivastava DK, Lehnert M, Paietta E, Pavelic ZP, Rimzsa L, Roninson IB, Sikic BI, Twentyman PR, Warnke R and Weinstein R (1996) Methods to detect P-glycoprotein-associated multidrug resistance: consensus recommendations. *Cancer Res* **56**: 3010–3020

Broxterman HJ, Schuurhuis GJ, Lankelma J, Baak JPA and Pinedo HM (1990) Towards functional screening for multidrug resistant cells in human malignancies. In *Pezcoller Foundation Symposium*, Vol. 1. *Drug Resistance: Mechanisms and Reversal*, Mihich E. (ed.), pp. 309–319. John Libbey CIC: Rome

Broxterman HJ, Feller N, Kuiper CM, Boven E, Versantvoort CHM, Teerlink T, Pinedo, HM and Lankelma J (1995) Correlation between functional and molecular analysis of mdrl P-glycoprotein in human solid tumor xenografts. *Int J Cancer* **61**: 880–886

Broxterman HJ, Lankelma J and Pinedo HM (1996*a*) How to probe clinical tumour samples for P-glycoprotein and multidrug resistance-associated protein. *Eur J Cancer* **32A**: 1024–1033

Broxterman HJ, Sonneveld P, Feller N, Ossenkoppele GJ, Währer DCR, Eekman CA, Schoester M, Lankelma J, Pinedo HM, Löwenberg B and Schuurhuis GJ (1996*b*) Quality control of multidrug resistance assays in adult acute leukemia: correlation between assays for P-glycoprotein expression and activity. *Blood* **86**: 4809–4816

Broxterman HJ, Lankelma J, Pinedo HM, Eekman CA, Währer DCR, Ossenkoppele GJ and Schuurhuis GJ (1997) Theoretical and practical considerations for the measurement of P-glycoprotein function in acute myeloid leukemia. *Leukemia* **11**: (in press)

Chaudhary PM and Roninson IB (1991) Expression and activity of P-glycoprotein, a multidrug efflux pump, in human hematopoietic stem cells. *Cell* **66**: 85–94

Dairkee SH, Deng G, Stampfer MR, Waldman FM and Smith HS (1995). Selective cell culture of primary breast carcinoma. *Cancer Res* **55**: 2516–2519

Feller N, Broxterman HJ, Währer DCR and Pinedo HM (1995*a*) ATP-dependent efflux of calcein by the multidrug resistance protein (MRP): no inhibition by intracellular glutathione depletion. *FEBS Lett* **368**: 385–388

Feller N, Kuiper CM, Lankelma J, Ruhdal JK, Scheper RJ, Pinedo HM and Broxterman HJ (1995*b*) Functional detection of *MDR*1/P170 and *MRP*/P190-mediated multidrug resistance in tumour cells by flow cytometry. *Br J Cancer* **72**: 543–549

Ino T, Miyazaki H, Isogai M, Nomura T, Tsuzuki M, Tsuruo T, Ezaki K and Hirano M (1994) Expression of P-glycoprotein in *de novo* acute myelogenous leukemia at initial diagnosis: results of molecular and functional assays, and correlation with treatment outcome. *Leukemia* **8**: 1492–1497

Juliano, RL and Ling V (1976) A surface glycoprotein modulating drug permeability in Chinese hamster ovary cell mutants. *Biochem Biophys Acta* **455**: 152–162

Kartner N, Evernden-Porelle D, Bradley G and Ling V (1985) Detection of P-glycoprotein in multidrug-resistant cell lines by monoclonal antibodies. *Nature* **316**: 820–823

Kunikane H, Zalupski MM, Kukuruga M, Lucas DR, Ryan JR, Krishan A and Vaitkevicius V (1995) P-glycoprotein expression and drug efflux in soft tissue sarcoma. *Proc Am Assoc Cancer Res* **36**: 219

Leith CP, Chen I-M, Kopecky KJ, Appelbaum FR, Head DR, Godwin JE, Weick JK and Willman CL (1995) Correlation of multidrug resistance (MDR1) protein expression with functional dye/drug efflux in acute myeloid leukemia by multiparameter flow cytometry: identification of discordant MDR-/efflux+ and MDR+/efflux- cases. *Blood* **86**: 2329–2342

Ljung B-M, Mayall B, Lottich C, Boyer C, Sylvester SS, Leight GS, Siegler HF and Smith HS (1989) Cell dissociation techniques in human breast cancer – variations in tumor cell viability and DNA ploidy. *Breast Cancer Res Treat* **13**: 153–159

Marie J-P, Faussat-Suberville A-M, Zhou D and Zittoun R (1992) Daunorubicin uptake by leukemic cells: correlations with treatment outcome and *mdr*1 expression. *Leukemia* **7**: 825–831

Neyfakh AA, Serpinskaya AS, Chervonsky AV, Apasov SG and Kazarov AR (1989) Multidrug resistance phenotype of a subpopulation of T-lymphocytes without drug selection. *Exp Cell Res* **185**: 496–505

Noonan KE, Beck C, Holzmayer TA, Chin JE, Wunder JS, Andrulis IL, Gazdar AF, Willman CL, Griffith B, Von Hoff DD and Roninson IB (1990) Quantitative analysis of *MDR*1 (multidrug resistance) gene expression in human tumors by polymerase chain reaction. *Proc Natl Acad Sci USA* **87**: 7160–7164

Ross DD, Wooten PJ, Sridhara R, Ordóñez JV, Lee EJ and Schiffer CA (1993) Enhancement of daunorubicin accumulation, retention, and cytotoxicity by verapamil or cyclosporin A in blast cells from patients with previously untreated acute myeloid leukemia. *Blood* **82**: 1288–1299

Scheper RJ, Bulte JWM, Brakkee JGP, Quak JJ, Van Der Schoot E, Balm AJM, Meijer CJLM, Broxterman HJ, Kuiper CM, Lankelma J and Pinedo HM (1988) Monoclonal antibody JSB-1 detects a highly conserved epitope on the P-glycoprotein associated with multidrug resistance. *Int J Cancer* **42**: 389–394

Spoelstra EC, Westerhoff HV, Dekker H and Lankelma J (1992) Kinetics of daunorubicin transport by P-glycoprotein of intact cancer cells. *Eur J Biochem* **207**: 567–579

Willingham MC, Cornwell MM, Cardarelli CO, Gottesman MM and Pastan I (1986) Single cell analysis of daunomycin uptake and efflux in multidrug-resistant and-sensitive KB cells: effects of verapamil and other drugs. *Cancer Res* **46**: 5941–5946

© Cancer Research Campaign 1997


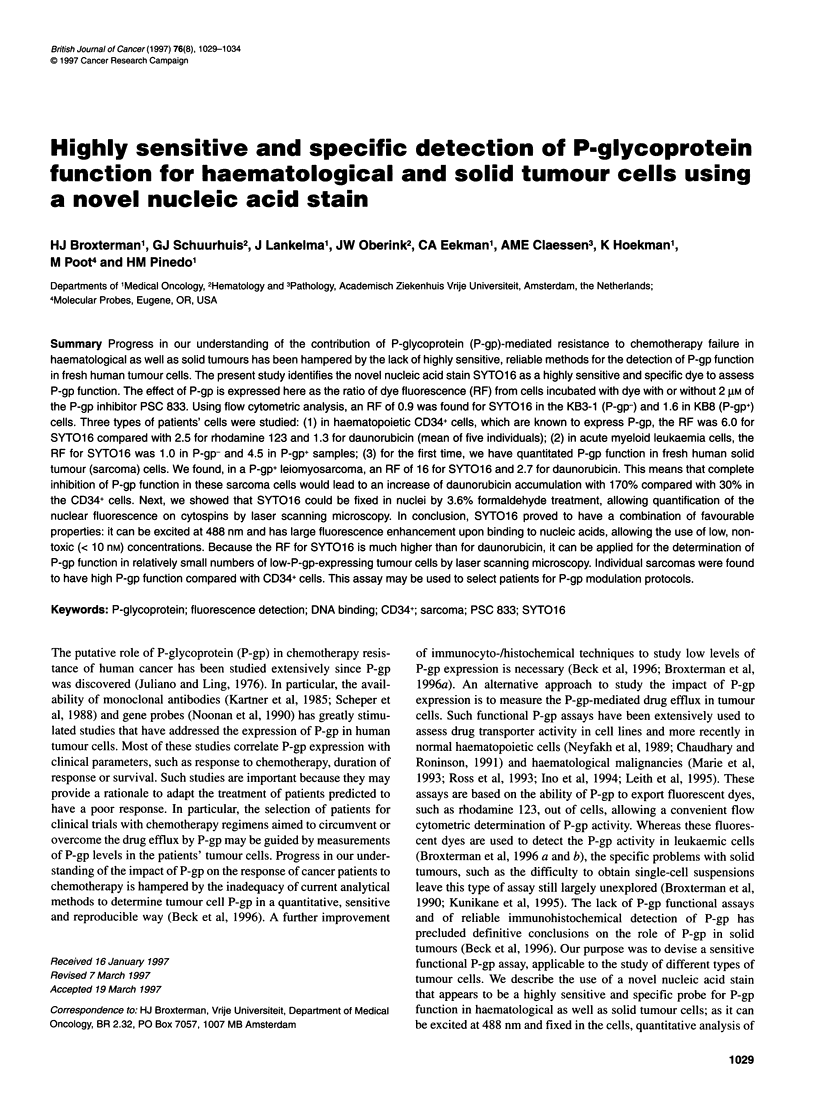

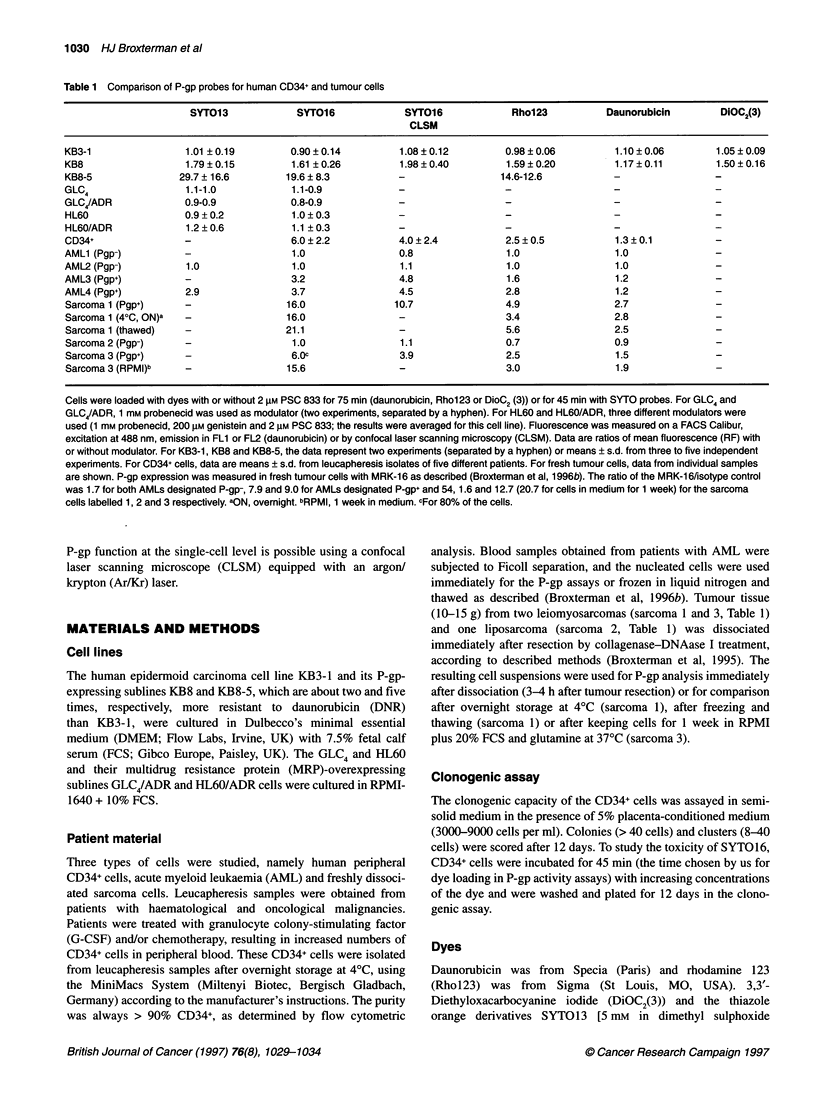

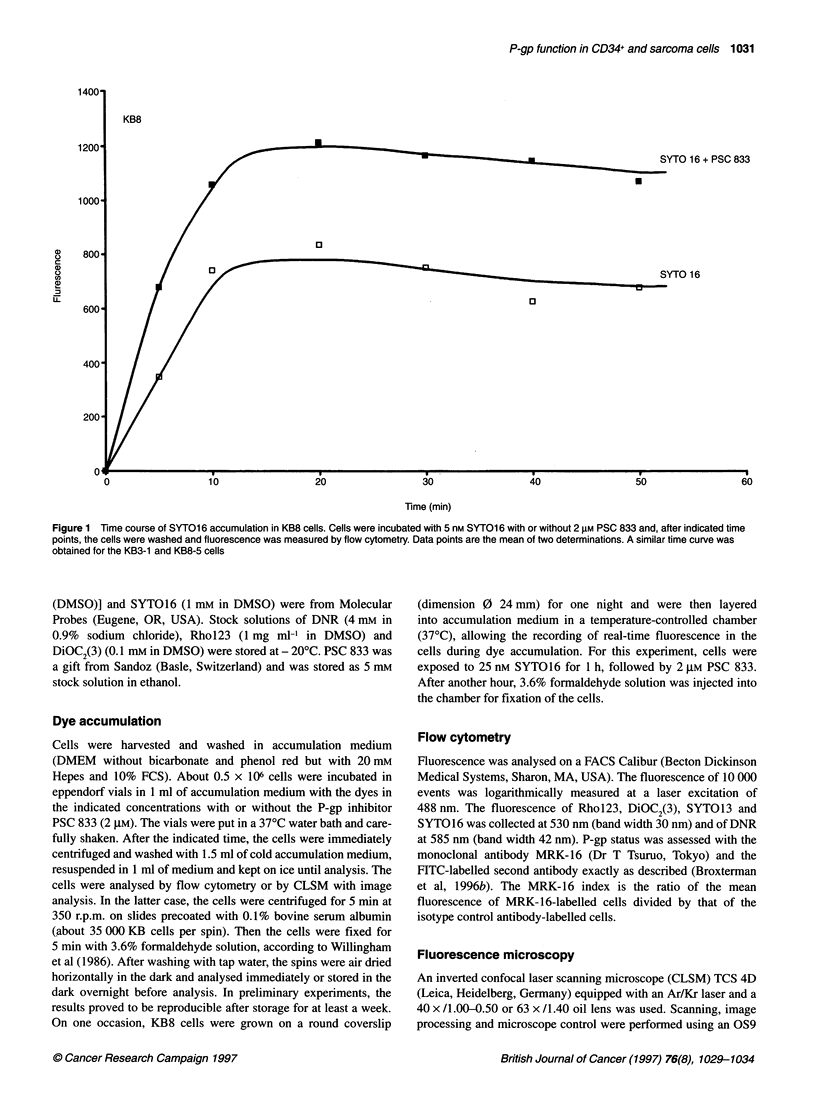

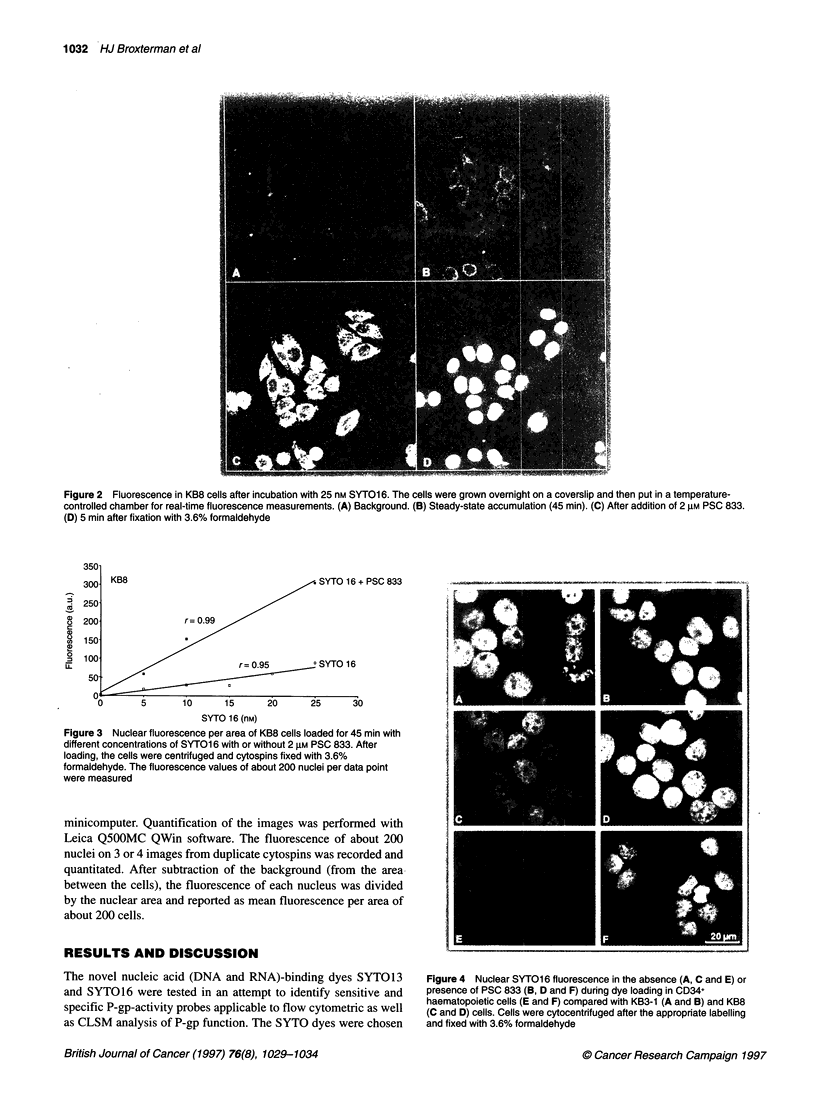

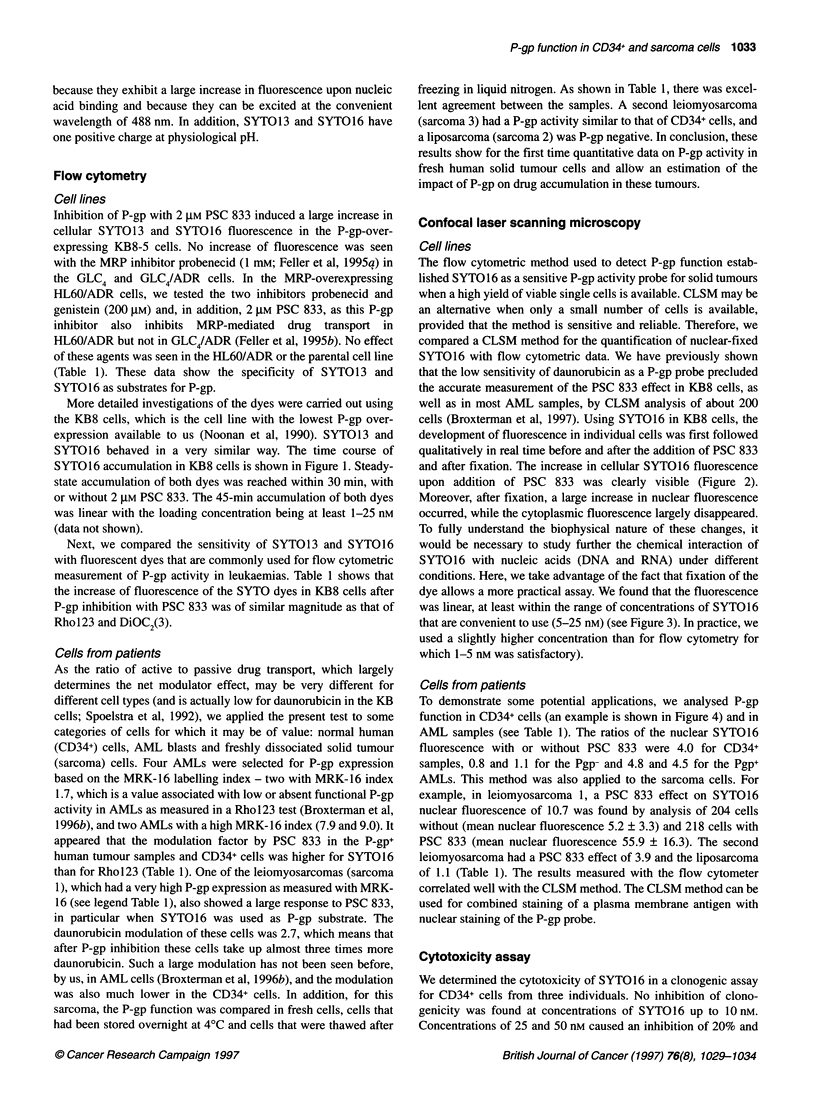

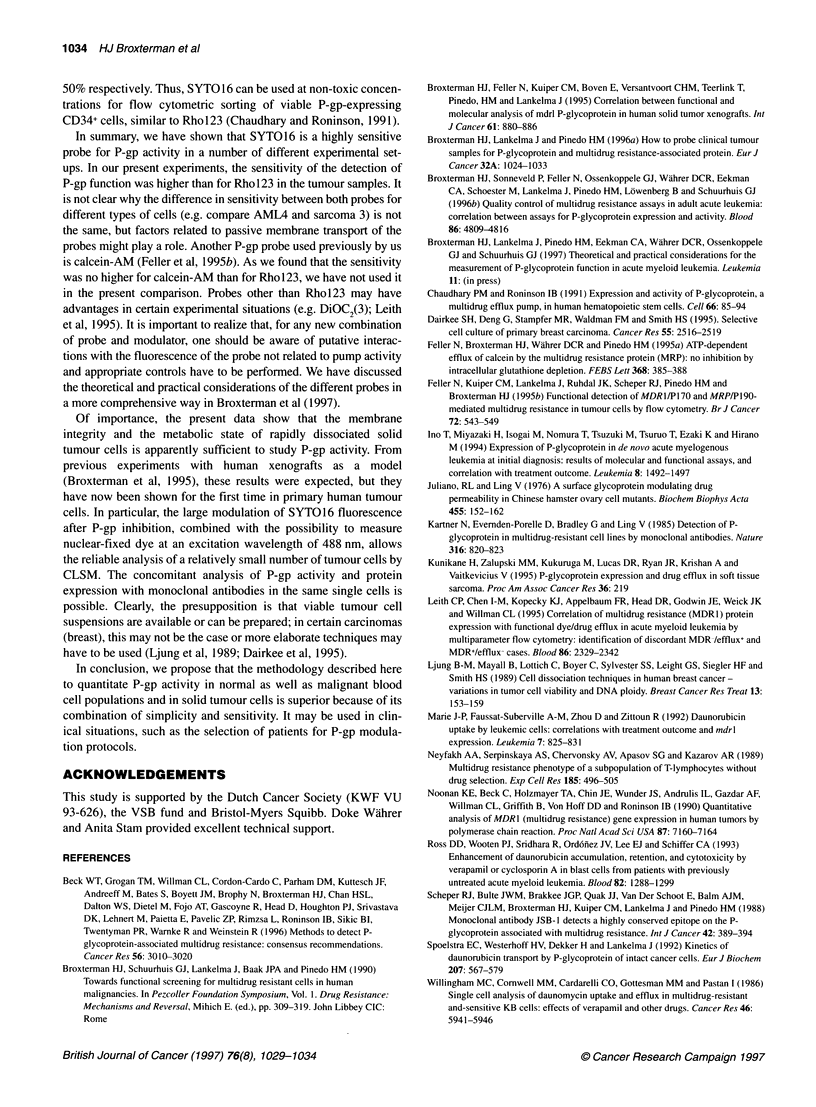


## References

[OCR_00562] Beck W. T., Grogan T. M., Willman C. L., Cordon-Cardo C., Parham D. M., Kuttesch J. F., Andreeff M., Bates S. E., Berard C. W., Boyett J. M. (1996). Methods to detect P-glycoprotein-associated multidrug resistance in patients' tumors: consensus recommendations.. Cancer Res.

[OCR_00579] Broxterman H. J., Feller N., Kuiper C. M., Boven E., Versantvoort C. H., Teerlink T., Pinedo H. M., Lankelma J. (1995). Correlation between functional and molecular analysis of mdr1 P-glycoprotein in human solid-tumor xenografts.. Int J Cancer.

[OCR_00588] Broxterman H. J., Lankelma J., Pinedo H. M. (1996). How to probe clinical tumour samples for P-glycoprotein and multidrug resistance-associated protein.. Eur J Cancer.

[OCR_00593] Broxterman H. J., Sonneveld P., Feller N., Ossenkoppele G. J., Währer D. C., Eekman C. A., Schoester M., Lankelma J., Pinedo H. M., Löwenberg B. (1996). Quality control of multidrug resistance assays in adult acute leukemia: correlation between assays for P-glycoprotein expression and activity.. Blood.

[OCR_00608] Chaudhary P. M., Roninson I. B. (1991). Expression and activity of P-glycoprotein, a multidrug efflux pump, in human hematopoietic stem cells.. Cell.

[OCR_00612] Dairkee S. H., Deng G., Stampfer M. R., Waldman F. M., Smith H. S. (1995). Selective cell culture of primary breast carcinoma.. Cancer Res.

[OCR_00621] Feller N., Kuiper C. M., Lankelma J., Ruhdal J. K., Scheper R. J., Pinedo H. M., Broxterman H. J. (1995). Functional detection of MDR1/P170 and MRP/P190-mediated multidrug resistance in tumour cells by flow cytometry.. Br J Cancer.

[OCR_00627] Ino T., Miyazaki H., Isogai M., Nomura T., Tsuzuki M., Tsuruo T., Ezaki K., Hirano M. (1994). Expression of P-glycoprotein in de novo acute myelogenous leukemia at initial diagnosis: results of molecular and functional assays, and correlation with treatment outcome.. Leukemia.

[OCR_00634] Juliano R. L., Ling V. (1976). A surface glycoprotein modulating drug permeability in Chinese hamster ovary cell mutants.. Biochim Biophys Acta.

[OCR_00639] Kartner N., Evernden-Porelle D., Bradley G., Ling V. Detection of P-glycoprotein in multidrug-resistant cell lines by monoclonal antibodies.. Nature.

[OCR_00649] Leith C. P., Chen I. M., Kopecky K. J., Appelbaum F. R., Head D. R., Godwin J. E., Weick J. K., Willman C. L. (1995). Correlation of multidrug resistance (MDR1) protein expression with functional dye/drug efflux in acute myeloid leukemia by multiparameter flow cytometry: identification of discordant MDR-/efflux+ and MDR1+/efflux- cases.. Blood.

[OCR_00657] Ljung B. M., Mayall B., Lottich C., Boyer C., Sylvester S. S., Leight G. S., Siegler H. F., Smith H. S. (1989). Cell dissociation techniques in human breast cancer--variations in tumor cell viability and DNA ploidy.. Breast Cancer Res Treat.

[OCR_00664] Marie J. P., Faussat-Suberville A. M., Zhou D., Zittoun R. (1993). Daunorubicin uptake by leukemic cells: correlations with treatment outcome and mdr1 expression.. Leukemia.

[OCR_00669] Neyfakh A. A., Serpinskaya A. S., Chervonsky A. V., Apasov S. G., Kazarov A. R. (1989). Multidrug-resistance phenotype of a subpopulation of T-lymphocytes without drug selection.. Exp Cell Res.

[OCR_00674] Noonan K. E., Beck C., Holzmayer T. A., Chin J. E., Wunder J. S., Andrulis I. L., Gazdar A. F., Willman C. L., Griffith B., Von Hoff D. D. (1990). Quantitative analysis of MDR1 (multidrug resistance) gene expression in human tumors by polymerase chain reaction.. Proc Natl Acad Sci U S A.

[OCR_00681] Ross D. D., Wooten P. J., Sridhara R., Ordóez J. V., Lee E. J., Schiffer C. A. (1993). Enhancement of daunorubicin accumulation, retention, and cytotoxicity by verapamil or cyclosporin A in blast cells from patients with previously untreated acute myeloid leukemia.. Blood.

[OCR_00687] Scheper R. J., Bulte J. W., Brakkee J. G., Quak J. J., van der Schoot E., Balm A. J., Meijer C. J., Broxterman H. J., Kuiper C. M., Lankelma J. (1988). Monoclonal antibody JSB-1 detects a highly conserved epitope on the P-glycoprotein associated with multi-drug-resistance.. Int J Cancer.

[OCR_00693] Spoelstra E. C., Westerhoff H. V., Dekker H., Lankelma J. (1992). Kinetics of daunorubicin transport by P-glycoprotein of intact cancer cells.. Eur J Biochem.

[OCR_00698] Willingham M. C., Cornwell M. M., Cardarelli C. O., Gottesman M. M., Pastan I. (1986). Single cell analysis of daunomycin uptake and efflux in multidrug-resistant and -sensitive KB cells: effects of verapamil and other drugs.. Cancer Res.

